# Complete Genome Sequence of a Novel Nonnodulating *Rhizobium* Species Isolated from *Agave americana* L. Rhizosphere

**DOI:** 10.1128/genomeA.01280-17

**Published:** 2017-11-16

**Authors:** Víctor Manuel Ruíz-Valdiviezo, Marco Antonio Rogel-Hernandez, Gabriela Guerrero, Clara Ivette Rincón-Molina, Luis Galdino García-Perez, Federico Antonio Gutiérrez-Miceli, Juan José Villalobos-Maldonado, Aline López-López, Esperanza Martinez-Romero, Reiner Rincón-Rosales

**Affiliations:** a Instituto Tecnológico de Tuxtla Gutiérrez, Tecnológico Nacional de México, Tuxtla Gutiérrez, México; b Centro de Ciencias Genómicas, Universidad Nacional Autónoma de México, Cuernavaca, Morelos, México; c Universidad Autónoma de Tlaxcala, Tlaxcala-Texmelucan, Ixtacuixtla, Tlaxcala, México

## Abstract

We report here the complete genome sequence of *Rhizobium* sp. strain ACO-34A, isolated from *Agave americana* L. rhizosphere. No common *nod* genes were found, but there were *nif* genes for nitrogen fixing. A low average nucleotide identity to reported species supports its designation as a novel *Rhizobium* species that has a complete ribosomal operon in a plasmid.

## GENOME ANNOUNCEMENT

Nitrogen-fixing microorganisms and plant growth-promoting bacteria are useful alternatives for replacing chemical fertilizers in nonlegume plants (1). *Rhizobium* sp. strain ACO-34A was isolated from *Agave americana* L. rhizosphere and promotes plant growth due to phosphate solubilization and indole-3-acetic acid biosynthesis (2). We found that ACO-34A does not form nodules in *Phaseolus vulgaris*, which is a promiscuous plant for nodulation.

The ACO-34A genome was sequenced using the Pacific Biosciences (PacBio RSII) single-molecule real-time (SMRT) platform. Three SMRT cells of a 15- to 20-kb insert library were sequenced; 271,302 reads, with an average read length of 11,950 bp, were used for the *de novo* genome assembly using RS_HGAP_Assembly.3 for SMRT Portal Analysis version 2.3.0 (3), and a 347× coverage was obtained. Genome annotation was performed using the NCBI Prokaryotic Genome Annotation Pipeline (http://www.ncbi.nlm.nih.gov/genome/annotation_prok). Clusters of orthologous groups (4) were allocated using BLASTx searches, and hits with an *E* value cutoff of 1e^−10^ were considered. rRNA operons and prophages were identified with RNAmmer (5) and PHAST (6), respectively. Average nucleotide identity (ANI) (7) was calculated with the pyani module of Python (8). Relative synonymous codon usage and principal component analysis were applied to identify chromids and plasmids (9).

The 6,284,736-bp (61% GC content) ACO-34A genome sequence comprises one chromosome (4.75 Mb) and four extrachromosomal replicons that correspond to two plasmids (516 and 213 kb) and two chromids (494 and 305 kb) according to reported standards (9). The 516-kb plasmid contains one complete operon of ribosomal genes identical to the three chromosomal homologs. Ribosomal genes located on a plasmid may be the result of a transposition-integration event since they are contiguous to an integrase gene. In addition, *repA* and *repB* phylogenies group each of the extrachromosomal replicons with respective chromids or plasmids. In other rhizobia, genes for nodulation and biological nitrogen fixation are located in symbiotic plasmids (pSym) with sizes from 370 to 600 kb (10, 11). The 213-kb plasmid from strain ACO-34A might be a deleted pSym because it lacks *nod* genes, although it contains the *nifHDK* operon that encodes nitrogenase structural genes and other *nif* and *fix* genes; this plasmid is smaller than other pSyms and could be conjugative because it contains *tra* and *trb* genes. Host specificity genes, such as the* nodL*, *nodT*, and *nodN*, were found in the chromosome (as in *R. etli* CFN42 and *R. gallicum* R602). A 57.7-kb chromosomal region represents a complete prophage with 80 open reading frames, 34 of which resembled *Rhizobium* phage RR1-A (NCBI reference sequence NC_021560), a temperate phage found in deep subseafloor sediments (12). The ANI values in comparison to 221 *Rhizobium* genomes showed 85% identity to *R. selenitireducens* ATCC BAA-1503 (a nonnodulating environmental isolate) (13), but the ANI values were lower in comparison to other species, suggesting that ACO-34A corresponds to a new species, which could be named *R. agavense*. The genomic information for *Rhizobium* sp. ACO-34A may be used to further explore the role of nonnodulating rhizobia in promoting growth in nonlegume plants.

### Accession number(s).

The whole-genome nucleotide sequence of *Rhizobium* sp. ACO-34A was deposited in GenBank under the accession numbers CP021371, CP021372, CP021373, CP021374, and CP021375.
